# Subclinical Magnetic Resonance Imaging Markers of Cerebral Small Vessel Disease in Relation to Office and Ambulatory Blood Pressure Measurements

**DOI:** 10.3389/fneur.2022.908260

**Published:** 2022-07-14

**Authors:** Jesus D. Melgarejo, Gladys E. Maestre, Jose Gutierrez, Lutgarde Thijs, Luis J. Mena, Ciro Gaona, Reinier Leendertz, Joseph H. Lee, Carlos A. Chávez, Gustavo Calmon, Egle Silva, Dongmei Wei, Joseph D. Terwilliger, Thomas Vanassche, Stefan Janssens, Peter Verhamme, Daniel Bos, Zhen-Yu Zhang

**Affiliations:** ^1^Research Unit Hypertension and Cardiovascular Epidemiology, KU Leuven Department of Cardiovascular Sciences, Studies Coordinating Center, KU Leuven, Leuven, Belgium; ^2^Laboratory of Neurosciences, Faculty of Medicine, University of Zulia, Maracaibo, Venezuela; ^3^Department of Neurosciences and Department of Human Genetics, University of Texas Rio Grande Valley School of Medicine, Brownsville, TX, United States; ^4^Alzheimer's Disease Resource Center for Minority Aging Research, University of Texas Rio Grande Valley, Brownsville, TX, United States; ^5^South Texas ADRC, Laredo, TX, United States; ^6^Department of Neurology, College of Physicians and Surgeons, Columbia University, New York, NY, United States; ^7^Department of Informatics, Universidad Politécnica de Sinaloa, Mazatlán, Mexico; ^8^Taub Institute for Research in Alzheimer's Disease and the Aging Brain and the G.H. Sergievsky Center at Columbia University Medical Center, New York, NY, United States; ^9^Departments of Epidemiology and Neurology, Columbia University Medical Center, New York, NY, United States; ^10^Laboratory of Ambulatory Recordings, Cardiovascular Institute (IECLUZ), University of Zulia, Maracaibo, Venezuela; ^11^Department of Genetics and Development, Department of Psychiatry, and G.H. Sergievsky Center, Columbia University, New York, NY, United States; ^12^Division of Medical Genetics, New York State Psychiatric Institute, New York, NY, United States; ^13^Division of Public Health Solutions, National Institute for Health and Welfare, Helsinki, Finland; ^14^Center for Molecular and Vascular Biology, KU Leuven Department of Cardiovascular Sciences, KU Leuven, Leuven, Belgium; ^15^Division of Cardiology, Department of Internal Medicine, University Hospitals UZ Leuven, Leuven, Belgium; ^16^Department of Epidemiology, Erasmus Medical Center, Rotterdam, Netherlands; ^17^Department of Radiology and Nuclear Medicine, Erasmus Medical Center, Rotterdam, Netherlands

**Keywords:** cerebral small vessel disease, white matter hyperintensities, lacunes, cerebral microbleeds, enlarged perivascular spaces, ambulatory blood pressure monitoring, nighttime blood pressure

## Abstract

**Background:**

Twenty-four-hour and nighttime blood pressure (BP) levels are more strongly associated with cardiovascular risk than office or daytime BP measurements. However, it remains undocumented which of the office and ambulatory BP measurements have the strongest association and predictive information in relation to the presence of type I, or arteriolosclerosis type, cerebral small vessel diseases (CSVD).

**Methods:**

A subset of 429 participants from the Maracaibo Aging Study [aged ≥40 years (women, 73.7%; mean age, 59.3 years)] underwent baseline brain magnetic resonance imaging (MRI) to visualize CSVD, which included log-transformed white matter hyperintensities (log-WMH) volume and the presence (yes/no) of lacunes, cerebral microbleeds (CMB), or enlarged perivascular spaces (EPVS). Linear and logistic regression models were applied to examine the association between CSVD and each +10-mmHg increment in the office and ambulatory systolic BP measurements. Improvement in the fit of nested logistic models was assessed by the log-likelihood ratio and the generalized *R*^2^ statistic.

**Results:**

Office and ambulatory systolic BP measurements were related to log-WMH (β*-*correlation coefficients ≥0.08; *P* < 0.001). Lacunes and CMB were only associated with ambulatory systolic BP measurements (odds ratios [OR] ranged from 1.31 [95% confidence interval, 1.10-1.55] to 1.46 [1.17–1.84], *P* ≤ 0.003). Accounted for daytime systolic BP, both the 24-h (β-correlation, 0.170) and nighttime (β*-*correlation, 0.038) systolic BP measurements remained related to log-WMH. When accounted for 24-h or daytime systolic BP levels, the nighttime systolic BP retained the significant association with lacunes (ORs, 1.05–1.06; 95% CIs, ≥1.01 to ≤ 1.13), whereas the 24-h and daytime systolic BP levels were not associated with lacunes after adjustments for nighttime systolic BP (ORs, ≤ 0.88; 95% CI, ≥0.77 to ≤ 1.14). On top of covariables and office systolic BP, ambulatory systolic BP measurements significantly improved model performance (1.05% ≥ *R*^2^ ≤ 3.82%). Compared to 24-h and daytime systolic BP, nighttime systolic BP had the strongest improvement in the model performance; for WMH (1.46 vs. 1.05%) and lacunes (3.06 vs. ≤ 2.05%).

**Conclusions:**

Twenty-four-hour and nighttime systolic BP were the more robust BP measurements associated with CSVD, but the nighttime systolic BP level had the strongest association. Controlling ambulatory BP levels might provide additional improvement in the prevention of CSVD.

## Introduction

Cerebral small vessel disease (CSVD) has repeatedly been linked to poor cognition, increased risk of stroke, and dementia (1, 2). Arteriolosclerosis or type I CSVD is the most common type of CSVD and includes white matter hyperintensities (WMH), lacunes, cerebral microbleeds, and enlarged perivascular spaces (3). Within the etiological framework of type I CSVD, hypertension plays an essential role. Numerous studies have documented that the optimal control of blood pressure (BP) level significantly reduced the progression of these lesions (4–6), which, in turn, lowers the risk for adverse health outcomes associated with aging (7, 8). To monitor BP control through antihypertensive therapy, adequate measurement of the BP is imperative (9). Current guidelines recognize that ambulatory BP monitoring is the state-of-art method to quantify the BP level compared to office BP measurements (10, 11), and 24-h and nighttime BP levels have the strongest association with cardiovascular outcomes compared to office and daytime BP levels (12). Office and ambulatory BP measurements are individually associated with the presence and development of CSVD in population-based studies (13–15) and patient cohorts (16–20). Nevertheless, no studies have been compared, which BP measurements have the strongest association with CSVD, especially, for lacunes, cerebral microbleeds, and enlarged perivascular spaces in the general population (13–15). Moreover, it remains undocumented whether ambulatory BP measurements provide additional information for the prediction of CSVD beyond office BP and conventional risk factors. To address these issues, we analyzed the Maracaibo Aging Study data to assess the association of subclinical markers of CSVD with office and ambulatory BP measurements. We also tested the improvement of the model performance of ambulatory BP measurements on top of office BP and conventional risk factors in the prediction of CSVD.

## Materials and Methods

### Study Cohort

The Maracaibo Aging Study is a prospective population-based study of community-dwelling individuals ≥40 years of age from the Santa Lucia (since 1998) and Santa Rosa de Agua (since 2010) neighborhoods located in the city of Maracaibo, Venezuela (21). Detailed methodology of the Maracaibo Aging Study is described elsewhere (21). From August 2011 until June 2016, 534 participants were additionally enrolled, using a sampling frame prioritizing the female ancestral lines with the intention to engage in genetic studies. In this study phase, participants underwent neurological and cardiovascular assessments. The Institutional Review Boards of the Cardiovascular Institute at the University of Zulia, Maracaibo, and Columbia University, New York, approved the Maracaibo Aging Study, which complied with the Helsinki declaration for investigations into human subjects (22). All participants signed an informed consent form. For this study, we included participants who had ambulatory BP monitoring with a minimum number of 15/5 daytime and nighttime ambulatory BP recordings (10), and MRI scans evaluated between 2011 and 2016.

### Magnetic Resonance Imaging (MRI)

Magnetic resonance imaging (MRI) scans were obtained on a 1.5-T scanner (GE Healthcare). It included T1-weighted, T2-weighted, gradient echo, diffusion-weighted imaging, angiography, and, for the purposes of this study, T2-weighted fluid-attenuated inversion recovery (FLAIR) scans. The FLAIR image parameters were TR = 8,000 ms, TE = 123 ms, 2,000 [ms] inversion time, 25-cm FOV, 2 NEX, 256 × 192 matrix with 2-mm slice thickness, 0 mm spacing, 63 slices; 6:01; COIL 8NHEAD_A, and an oblique orientation (axial following commissural plane). Individuals with a pacemaker, aneurysm clip, neurostimulator, cochlear implant, or body weight >110 kg were excluded from MRI scans. The MRI images were transferred to Columbia University for morphometric analysis.

### Cerebral Small Vessel Disease

Subclinical CSVD was categorized into quantitative (total WMH) and qualitative (presence of lacunes, cerebral microbleeds, and enlarged perivascular spaces) measurements (23). *WMH* were automatically quantified as hyperintensities observed on T2-weighted MRI. The full description of how WMH are automatically quantified has been published elsewhere (24). Briefly, each participant's T2-weighted FLAIR image was brain-extracted, and a single Gaussian curve was fit to voxel intensity values in the brain-extracted image. Intensity values of >2.1 standard deviations (SD) above the whole-brain mean intensity value were labeled as hyperintense voxels. The threshold was set by a trained operator who visually inspected the results. After visual inspection of each image, and correction, if necessary, the total WMH volume was derived by summing the number of labeled voxels and multiplying the value by the voxel dimensions. The WMH volumes were co-registered to the brain-extracted T1-weighted volumes defined by FreeSurfer (version 6.0, surfer.nmr.mgh.harvard.edu), and the Euclidean distance between each WMH-labeled voxel was computed and accounted for total cranial volume. *Lacunes* were characterized as focal lesions of at least 3-mm diameter, with signal intensities corresponding to liquid (hyperintense on T2-weighted images and hypointense on FLAIR images). *Cerebral microbleeds* were defined as rounded hypointense lesions on T2-weighted gradient echo-images with a diameter of <10 mm, an additional visualization on T1-weighted scans for differential diagnosis against other microvascular lesions. Symmetrical hypointensities in the *globi pallidi*, likely representing calcifications, sulcal flow voids from cortical vessels, and hypointensities, possibly due to partial volume artifacts from bone, were disregarded. Cerebral microbleeds were identified as definite microbleeds based on the Microbleed Anatomical Rating Scale (25), and were classified as: total microbleeds and, also, based on their localization as lobar, deep, or mixed cerebral microbleeds. *Enlarged perivascular* or *Virchow*-*Robin spaces* were defined as round, oval, or linear-shaped lesions with a diameter of >5 mm, with a smooth margin, absence of mass effect, and with a signal intensity equal to interstitial fluid on T2-weighted images, with a flair rim around them, and (if visible) hypointense on FLAIR images without a hyperintense rim to distinguish them from old lacunar infarcts.

### Blood Pressure Measurement

A nurse or physician measured the office BP with a validated oscillometric device (Dynamap®). The office BP was the average of five consecutive readings in the sitting position. Validated oscillometric 90207 Spacelabs monitors (Snoqualmie, WA) (26) were programmed to obtain readings at 15-min intervals from 6 am until 11 pm, and at 30-min intervals from 11 pm until 6 am (Supplementary Table 1). The same SAS macro-processed all ambulatory BP recordings, which stayed unedited, with the exception of readings with higher diastolic BP than systolic BP, or flagged with an error code. The within-subject 24-h BP was a time-weighted average, giving weights to each individual reading proportional to the time lag with the previous reading. Hypertension categories based on office and ambulatory BP measurements were defined according to the ACC/AHA 2017 guidelines (11). Office hypertension was a systolic or diastolic BP of ≥130/80-mm Hg or use of antihypertensive drugs. 24-h, daytime, and nighttime hypertension were systolic or diastolic BP levels of ≥125/75, ≥130/80, and ≥110/65 mm Hg; respectively. The dipping ratio was calculated by dividing the nighttime by the daytime BP level (27).

### Demographic and Clinical Characteristics

We documented demographic and clinical information related to CSVD, and this was collected by physicians and trained nurses. The observers administrated questionnaires to collect each participant's medical history: smoking status, drinking habits, use of antihypertensive or antidiabetic medications, and educational attainment. History of smoking included past and current smoking. Obesity was defined as a body mass index (BMI) of ≥30 kg/m^2^. Diabetes mellitus was defined based on the criteria of the Seventh Report of the Joint National Committee (28) as a glucose serum level of ≥126 mg/dL or intake of anti-diabetic drugs. The glomerular filtration rate was estimated according to the method of the National Institute of Diabetes and Digestive and Kidney Disease (29). History of cardiovascular disease included ischemic coronary diseases, heart failure, or stroke prior to the MRI and BP assessments.

### Statistical Analyses

We compared means by *t*-tests or Wilcoxon-Mann-Whitney, and proportions by Fisher exact test. Due to a non-parametric distribution, we log-transformed total WMH (log-WMH) volume to conduct regression analyses and expressed associations as per +1 SD increment in the log-WMH. After stratification for sex, we interpolated missing values of BMI (*n* = 5), total serum cholesterol (*n* = 56), low-density (*n* = 79), and high-density serum cholesterol (*n* = 79), and serum creatinine (*n* = 60) from the regression slopes on age. We selected covariables based on their pathological role with CSVD (sex, age, BMI, diabetes mellitus, use of antihypertensive treatment, glomerular filtration rate, high-density serum cholesterol, and history of cardiovascular diseases) or potential confounders (education and cephalic circumference). We expressed odds ratios (for lacunes, cerebral microbleeds, and enlarged perivascular spaces) and correlation coefficients (for log-WMH) per +10/5-mm Hg increment in the systolic/diastolic BP, and per +0.10-mm Hg increment in the systolic dipping ratio. We estimated the probability of having CSVD according to categories of office, 24-h, daytime, and nighttime systolic BP (normal BP, elevated BP, stage-1, stage-2, and severe hypertension). The probability was derived from logistic regression models that accounted for the selected confounders, and heatmaps were constructed to visualize the contribution of each category to the probability of having CSVD. To estimate the probability for WMH, we had to divide log-WMH into normal (<90th of the percentile distribution) and high (≥90th) WMH.

To determine which office or ambulatory BP measurements were better associated with CSVD, we simultaneously included two BP measurements in the same model (e.g., 24-h and daytime BP). Because of the collinearity effect, we applied the residuals method to uncorrelated ambulatory BP measurements by regressing one measurement on the other. For example, when assessing whether nighttime BP was better associated with CSVD than 24-h BP, we regressed nighttime BP on 24-h BP and used the residuals to compute side effects (beta coefficients, odds ratios) while adjusting the model by 24-h BP. Improvement in the fit of nested logistic models was assessed by the log-likelihood ratio and the generalized *R*^2^ statistic. In sensitivity analyzes, we associated CSVD with office and ambulatory diastolic BP measurements, and we also conducted analyzes by excluding participants with previous strokes to ensure that the association between ambulatory BP levels and CSVD was not driven by the presence of stroke. A two-tailed α-level of ≤ 0.05 was deemed statistically significant. For database management and statistical analysis, we used SAS software, version 9.4, maintenance level five.

## Results

### Baseline Characteristics

The study population had a mean age of 59.3 (range, 5th-95th percentile interval, 49–70 years) and included 73.7% (*n* = 316) women (Table 1). Among 429 participants, one-third were smokers (28.7%), had obesity (32.4%), or were on antihypertensive treatment (*n* = 33.8%). The prevalence of diabetes mellitus, history of cardiovascular diseases, and stroke was 16.3, 7.5, and 2.6%, respectively. Almost half of the participants had an office (67.4%) or a 24-h (45.4%) hypertension, whereas the prevalence of daytime and nighttime hypertension was 35 and 68.1%, respectively. In addition, we reported 8.9% (*n* = 38) participants with lacunes, 11.9% (*n* = 51) with cerebral microbleeds, and 7.46% (*n* = 32) with enlarged perivascular spaces were detected (Table 1).

**Table 1:** Baseline characteristics of the Maracaibo Aging Study participants with brain MRI data.

**Characteristics**	**Whole sample (*n* = 429)**
**No of participants — no. (%)**	
Women — no. (%)	316 (73.7)
Smoking — no. (%)	123 (28.7)
Drinking alcohol — no. (%)	187 (45.1)
Obesity — no. (%)	139 (32.4)
Diabetes mellitus — no. (%)	70 (16.3)
Previous history of CV diseases — no. (%)	32 (7.5)
Previous stroke — no. (%)	11 (2.6)
Office hypertension — no. (%)	289 (67.4)
24-H hypertension — no. (%)	195 (45.4)
Daytime hypertension — no. (%)	150 (35.0)
Nighttime hypertension — no. (%)	292 (68.1)
On antihypertensive treatment — no. (%)	145 (33.8)
**Cerebral small vessel disease — no. (%)**	
Lacunes	38 (8.9)
Cerebral microbleeds*	51 (11.9)
Lobar microbleeds	25 (6.2)
Deep microbleeds	30 (7.4)
Mixed microbleeds	8 (2.1)
Enlarged perivascular spaces	32 (7.5)
**Mean (±SD) median (IQR) with characteristic**	
Age — y	59.3 ± 13.0
Education — y†	6 (3–11)
Body mass index — kg/m2	28.0 ± 5.5
Cephalic circumference — cm	55.1 ± 1.9
Serum fasting glucose, mg/dL	108.4 ± 37.6
Total serum cholesterol — mg/dL	197.8 ± 44.9
High-density lipoprotein serum cholesterol — mg/dL	44.7 ± 11.9
Low-density lipoprotein serum cholesterol — mg/dL	127.5 ± 37.2
Serum triglycerides — mg/dL†	131 (89–173)
Glomerular filtration rate — mL/min per 1.73m^2^	79.4 ± 22.8
Office systolic/diastolic BP— mm Hg	140.1 ± 24.6/77.7 ± 11.0
24-h systolic/diastolic BP — mm Hg	121.6 ± 15.3/71.8 ± 9.2
Daytime systolic/diastolic BP — mm Hg	123.5 ± 14.6/73.7 ± 9.1
Night-time systolic/diastolic BP — mm Hg	116.8 ± 17.5/67.2 ± 10.7
White matter hyperintensities — cm^3^	2.63 (1.80–4.05)

*SD, standard deviation; IQR, interquartile range; BP, blood pressure*.

*Three cerebral microbleeds were infratentorial (brainstem and cerebellum).

*^†^Years of education and serum triacylglycerides were presented as interquartile range as measure of spread*.

### Cerebral Small Vessel Disease and Blood Pressure Measurements

In univariate regression analyses (Table 2), the office and ambulatory BP measurements were all significantly associated with CSVD (*P* ≤ 0.041). In adjusted regression models (Table 2), each +10-mm Hg increment in the office systolic was associated with larger total log-WMH (β correlation coefficient per +1-SD larger log-WMH = 0.08; 95% confidence interval [CI], 0.05-0.12; *P* < 0.001). Similarly, the associations were retained for the 24-h, daytime, and nighttime systolic BP (β coefficients between 0.12 and 0.14; 95% CI, ≥08 ≤ 0.19, *P* < 0.001). The odds ratios (ORs) for lacunes and cerebral microbleeds per +10-mm Hg increment in the 24-h, daytime, and nighttime systolic BP ranged between 1.31 (95% CI, 1.10–1.55; *P* = 0.002) and 1.47 (95% 1.21–1.79; *P* < 0.001). Based on the locations of the cerebral microbleeds (Supplementary Table 2), office systolic BP was only associated with mixed microbleeds (OR, 1.79; 95% CI, 1.17–2.73; *P* = 0.006); whereas, the presence of lobar or deep cerebral microbleeds was significantly associated with 24-h, daytime, and nighttime systolic BP (OR ≥ 1.30; 95% CI ranged from 1.04 to ≤ 4.31; *P* ≤ 0.022). Every +5-mm Hg increment on the level of ambulatory diastolic BP measurements was significantly associated with larger log-WMH [β correlation coefficient ≥0.05; 95% CI, ≥0.01 to ≤ 0.10; *P* ≤ 0.033 (Supplementary Table 3)], and the presence of enlarged perivascular spaces [ORs ranged between 1.22 and 1.36; 95% CI, ≥1.02 to ≤ 1.70; *P* ≤ 0.030 (Supplementary Table 3)]. Our findings were consistent after excluding the participants with history of stroke (Supplementary Table 4).

**Table 2:** Association of quantitative and categorical subclinical markers of cerebral small vessel disease with office and ambulatory blood pressure measurements.

**BP measurements**	**Total Log-WMHs**		**Lacunes**		**Cerebral microbleeds**		**Enlarged perivascular**	
	**(per 1-SD increase)**		**(*****n*** **= 38)**		**(*****n*** **= 51)**		**spaces (*****n*** **= 18)**	
	**β coefficient (95% CI)**	***P***	**OR (95% CI)**	***P***	**OR (95% CI)**	***P***	**OR (95% CI)**	***P***
**Unadjusted models**								
Office systolic BP	0.13 (0.10, 0.16)	<0.001	1.27 (1.13–1.44)	<0.001	1.19 (1.07–1.33)	0.002	1.16 (1.02–1.33)	0.024
24-H systolic BP	0.21 (0.16, 0.26)	<0.001	1.54 (1.27–1.87)	<0.001	1.44 (1.20–1.71)	<0.001	1.29 (1.05–1.60)	0.016
Daytime systolic BP	0.20 (0.15, 0.26)	<0.001	1.52 (1.24–1.90)	<0.001	1.45 (1.20–1.74)	<0.001	1.33 (1.07–1.65)	0.011
Nighttime systolic BP	0.18 (0.14, 0.22)	<0.001	1.52 (1.28–1.80)	<0.001	1.36 (1.16–1.58)	<0.001	1.21 (1.01–1.45)	0.041
**Adjusted models**								
Office systolic BP	0.08 (0.05, 0.12)	<0.001	1.14 (0.98–1.33)	0.09	1.13 (0.98–1.29)	0.08	1.14 (0.97–1.35)	0.11
24-H systolic BP	0.14 (0.09, 0.19)	<0.001	1.46 (1.17–1.84)	<0.001	1.39 (1.14–1.70)	0.001	1.27 (0.99–1.62)	0.050
Daytime systolic BP	0.13 (0.08, 0.19)	<0.001	1.42 (1.13–1.80)	0.003	1.40 (1.14–1.72)	0.002	1.30 (1.01–1.70)	0.038
Nighttime systolic BP	0.12 (0.07, 0.16)	<0.001	1.47 (1.21–1.79)	<0.001	1.31 (1.10–1.55)	0.002	1.19 (0.96–1.48)	0.11

*OR, odds ratio; CI, confidence interval; BP, blood pressure; WMH, white matter hyperintensities*.

*β coefficients and odds ratios are given by each +10-mm Hg increase in the systolic BP. Adjusted models accounted sex, age, education, cephalic circumference, body mass index, diabetes mellitus, high-density serum cholesterol, use of antihypertensive treatment, glomerular filtration rate, and previous history of cardiovascular diseases. The association of cerebral microbleeds according to its location (lobar, deep, or mixed) is shown in Supplementary Table 2*.

**Figure 1:**
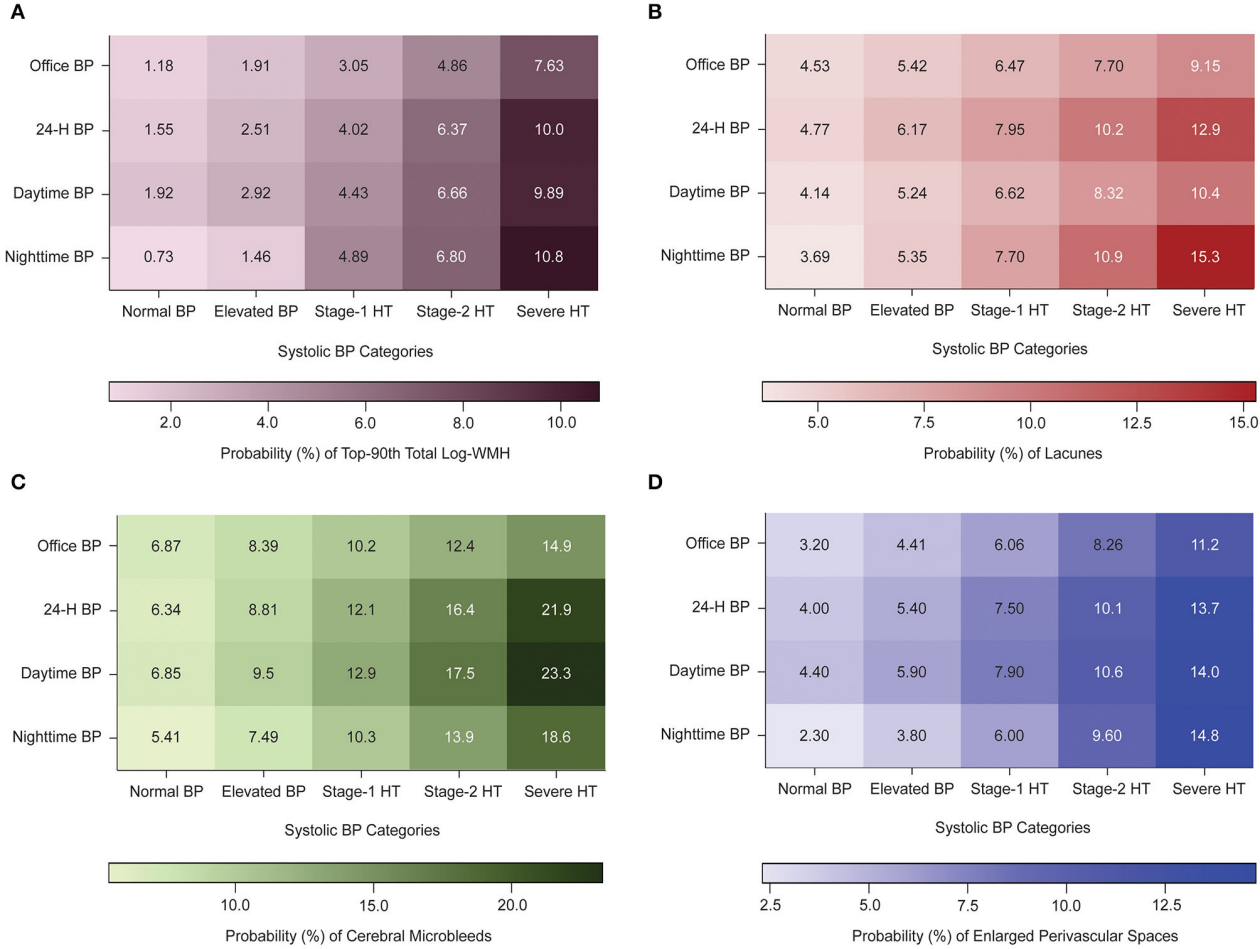
Heat maps displaying the probability (%) of having subclinical markers of cerebral small vessel disease according to office and ambulatory blood pressure Categories. BP indicates blood pressure; WMH, white matter hyperintensities; HT, hypertension. Normal, elevated BP, stage-1, stage-2, and severe hypertensions thresholds were 120/130/140/160 mm Hg for office systolic BP, 115/125/130/145 for 24-h, 120/130/135/145 for daytime, and 100/110/120/140 for nighttime BP. Numbers inside the box indicates the probability of having top-90th total log-WMH **(A)**, lacunes **(B)**, cerebral microbleeds **(C)**, or enlarged perivascular spaces **(D)**. The probability was derived by multivariable logistic regression and was standardized to the average of the distributions in the whole study population of sex, age, education, cephalic circumference, body mass index, diabetes mellitus, use of antihypertensive treatment, glomerular treatment, and history of cardiovascular diseases.

The probability of having a high volume of WMH and lacunes increased with nighttime systolic BP categories, ranging from 0.73–3.70% (normal BP, <100 mm Hg) to 10.8-15.3% (severe hypertension, ≥140 mm Hg), respectively (Figure 1). For these same categories, the probability according to office systolic BP categories ranged from 1.18 to 7.63%, and 4.53 to 9.15%. Similar trends were observed between ambulatory BP categories and other markers of CSVD.

### Comparison of Blood Pressure Measurements

**Figure 2:**
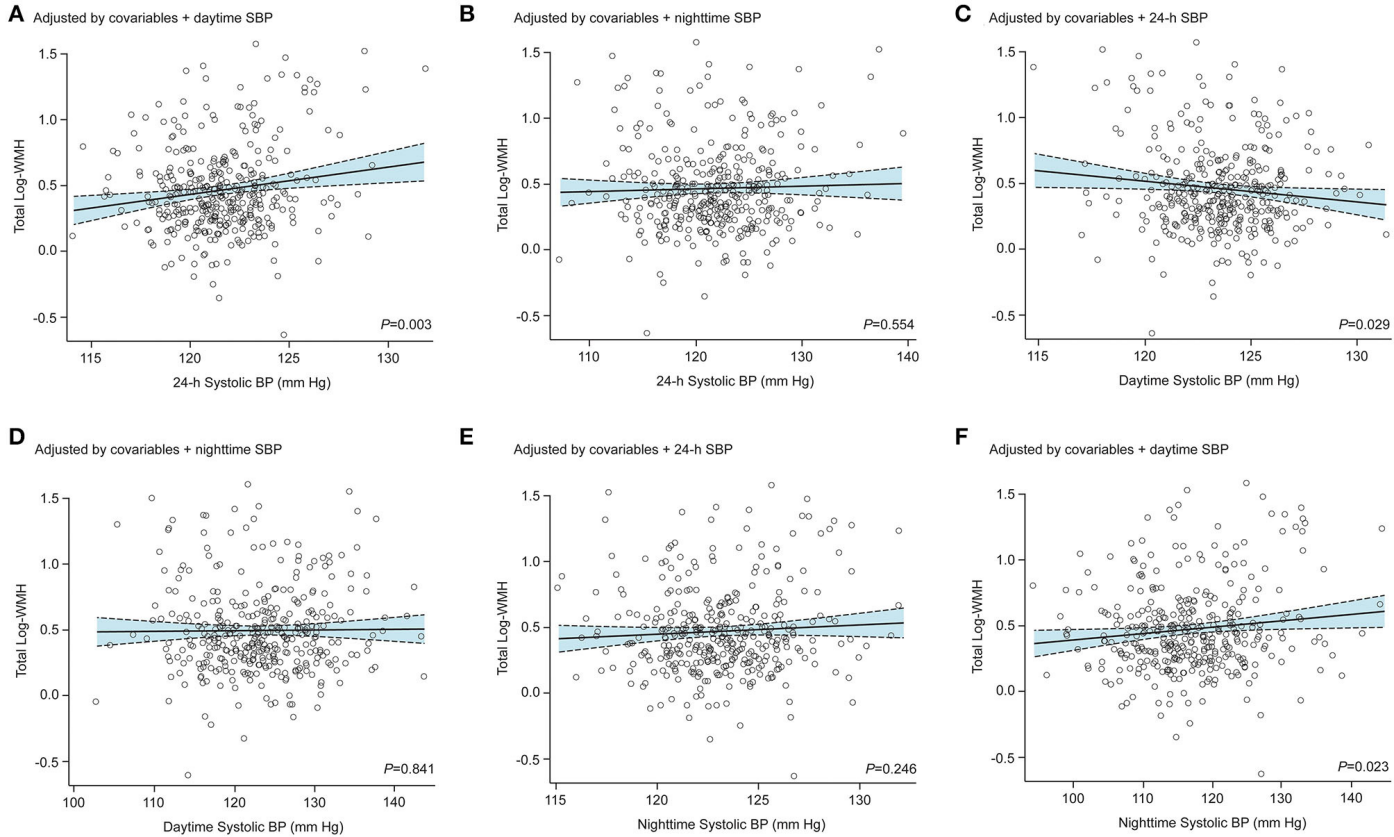
Association between total log-transformed white matter hyperintensities and standardized ambulatory systolic blood pressure measurements. The ambulatory blood pressure (BP) measurements were all standardized by sex, age, education, cephalic circumference, body mass index (BMI), diabetes mellitus, high-density serum cholesterol, use of antihypertensive treatment, glomerular filtration rate, and history of cardiovascular diseases. The 24-h systolic BP was additionally adjusted for daytime **(A)** and nighttime **(B)** systolic BP levels. The daytime systolic BP was additionally adjusted for 24-h **(C)** and nighttime **(D)** systolic BP levels. Finally, the nighttime systolic BP was additionally adjusted for 24-h **(E)** and daytime **(F)** systolic BP levels.

When adjusted for 24-h ambulatory BP measurements, the office systolic BP lost significance relationship with log-WMH (Supplementary Table 5; *P* ≥ 0.053). The ambulatory systolic BP measurements remained associated with log-WMH after adjustment for office BP (Supplementary Table 5; *P* ≤ 0.006). Adjustment for daytime systolic BP did not remove the statistical significance of 24-h and nighttime systolic BP in relation to total WMH (*P* ≤ 0.023), whereas the significance of higher daytime systolic BP level did not remain after adjustment for 24-h or nighttime systolic BP levels (Figure 2). For lacunes Table 3, nighttime systolic BP level retained the significance after accounted for 24-h or daytime systolic BP (ORs between 1.05 and 1.06; 95% CI ranged between ≥1.01 and ≤ 1.10; *P* ≤ 0.036). However, the 24-h (OR, 0.97; 95% CI, 0.91-1.04; *P* = 0.46) and daytime (OR, 0.88; 95% CI, 0.77–1.02; *P* = 0.09) systolic BP were not associated after adjustments for nighttime systolic BP. No association between ambulatory BP measurements and cerebral microbleeds (Table 3 and Supplementary Table 6), or enlarged perivascular spaces, (Table 3) were observed when these BP measurements were simultaneously included in the same logistic regression models (ORs ratios ranged from 0.96 to 1.10; 95% CI ranged from 0.84 to 1.23; *P* ≥ 0.17). After accounting for ambulatory 24-h or nighttime systolic BP measurements, the systolic dipping ratio was not associated with CSVD (Supplementary Table 7, *P* ≥ 0.18).

**Table 3:** Association between categorical subclinical markers of cerebral small vessel disease and ambulatory systolic blood pressure measurements.

**Quantitative markers of**	**Adjusted for**		**Adjusted for**		**Adjusted for**	
**cerebral small vessel disease**	**24-h systolic BP**		**daytime systolic BP**		**nighttime systolic BP**	
	**OR (95% CI)**	***P***	**OR (95% CI)**	***P***	**OR (95% CI)**	***P***
**Lacunes (*****n*** **=** **38)**						
24-H systolic BP	NA	NA	1.16 (1.02–1.33)	0.026	0.97 (0.91–1.04)	0.40
Daytime systolic BP	0.88 (0.77–1.02)	0.09	NA	NA	0.98 (0.94–1.03)	0.47
Nighttime systolic BP	1.06 (1.01–1.13)	0.036	1.05 (1.01–1.10)	0.001	NA	NA
**Cerebral microbleeds (*****n*** **=** **51)**						
24-H systolic BP	NA	NA	1.04 (0.92–1.16)	0.56	1.03 (0.97–1.09)	0.33
Daytime systolic BP	0.99 (0.88–1.13)	0.97	NA	NA	1.02 (0.98–1.07)	0.32
Nighttime systolic BP	1.01 (0.95–1.06)	0.91	1.01 (0.98–1.05)	0.53	NA	NA
**Enlarged perivascular spaces (*****n*** **=** **18)**						
24-H systolic BP	NA	NAA	0.96 (0.84–1.11)	0.60	1.04 (0.97–1.12)	0.20
Daytime systolic BP	1.07 (0.92–1.23)	0.38	NA	NA	1.03 (0.98–1.09)	0.17
Nighttime systolic BP	0.98 (0.93–1.04)	0.54	0.99 (0.95–1.04)	0.73	NA	NA

*OR, odds ratio; BP, blood pressure; WMH, white matter hyperintensities; NA, no applicable. OR are given by mm Hg unit increase in the systolic BP. Models accounted sex, age, education, cephalic circumference, body mass index, diabetes mellitus, high-density serum cholesterol, use of antihypertensive treatment, glomerular filtration rate, and previous history of cardiovascular diseases. Models including 2 correlated systolic BP measurements were constructed, using the residual method (see statistical analysis section). Office systolic BP did not remain associated with log-transformed total WMH after adjustment for 24-h systolic BP (P = 0.053, see Supplementary Table 6)*.

### Model Performance

The basic model accounted for sex, age, education, cephalic circumference, BMI, diabetes mellitus, use of antihypertensive treatment, glomerular filtration rate, and history of cardiovascular diseases (Table 4). On top of the basic model, office systolic BP significantly improved model performance for top-90th log-transformed WMH (*R*^2^ = 5.02%; *P* < 0.001), but not for lacunes, enlarged perivascular spaces, or cerebral microbleeds. In contrast, the inclusion of ambulatory systolic BP measurements on top of basic models and office systolic BP resulted in significant improvement of the model performance for top-90th log-WMH, lacunes, and cerebral microbleeds (*R*^2^ ranged from 0.97 to 3.09%; *P* ≥ 0.072).

**Table 4:** Nested fit of logistic models relating quantitative and categorical subclinical markers of cerebral small vessel disease and blood pressure measurements.

**Models**	**χ2 statistic**	***P***	***R*^2^ (%)†**
**Top-90th log-transformed-WMH (no/yes)**			
Base model*	64.3	<0.001	15.2
+ Office systolic BP	80.3	<0.001	4.00
+ Office systolic BP + 24-h systolic BP	84.4	0.042	1.05
+ Office systolic BP + Daytime systolic BP	83.0	0.098	0.69
+ Office systolic BP + Nighttime systolic BP	86.0	0.016	1.46
+ Office systolic BP + Ambulatory BP measurements	88.6	0.004	2.10
**Lacunes (no/yes)**			
Base model*	25.3	<0.001	6.27
+ Office systolic BP	28.5	0.074	0.81
+ Office systolic BP + 24-h systolic BP	36.6	0.004	2.05
+ Office systolic BP + Daytime systolic BP	34.4	0.015	1.50
+ Office systolic BP + Nighttime systolic BP	40.7	<0.001	3.06
+ Office systolic BP + Ambulatory BP measurements	43.8	<0.001	3.82
**Cerebral microbleeds (no/yes)**			
Base model*	16.5	<0.001	4.12
+ Office systolic BP	19.7	0.072	0.82
+ Office systolic BP + 24-h systolic BP	26.9	0.007	1.83
+ Office systolic BP + Daytime systolic BP	26.6	0.008	1.74
+ Office systolic BP + Nighttime systolic BP	25.9	0.012	1.59
+ Office systolic BP + Ambulatory BP measurements	27.0	0.007	1.86
**Enlarged perivascular spaces (no/yes)**			
Base model*	9.7	0.002	2.45
+ Office systolic BP	12.1	0.119	0.62
+ Office systolic BP + 24-h systolic BP	13.6	0.222	0.38
+ Office systolic BP + Daytime systolic BP	14.0	0.167	0.48
+ Office systolic BP + Nighttime systolic BP	12.8	0.405	0.18
+ Office systolic BP + Ambulatory BP measurements	15.7	0.057	0.92

*WMH denotes white matter hyperintensities, BP, blood pressure. On top of 24-h or nighttime systolic BP, office systolic BP only improved the model performance for top-90th log-transformed WMH (R^2^ from 1.02 to 1.14%; P = 0.045 and P = 0.034). When basic model additionally accounted for all the ambulatory systolic BP measurements, office systolic BP did not longer improve model performance (P = 0.074)*.

*Basic models accounted for sex, age, education, cephalic circumference, body mass index, diabetes mellitus, high-density serum cholesterol, use of antihypertensive treatment, glomerular filtration rate, and history of cardiovascular diseases.

*^†^R^2^ is an estimate of the additional variance explained (https://apha.confex.com/apha/134am/techprogram/paper_135906.htm)*.

## Discussion

In this population-based study, higher 24-h and nighttime systolic BPs, compared with office and daytime BP measurements, were better associated with a larger accumulation of WMH volume and a higher probability of having lacunes or cerebral microbleeds. The daytime BP, rather than nighttime BP, was better associated with enlarged perivascular spaces. The probability (%) of high burden of WMH volume and lacunes were higher across nighttime systolic BP categories compared to other BP measurements, with corresponding rates of 0.73–15.3% between normal systolic BP and severe systolic hypertension. In adjusted regression models, office systolic BP was only related with total WMH volume. However, this association did not remain significant after accounting for ambulatory BP measurements. Our findings were consistent in participants stroke-free history. The model performance was significantly improved by 24-h and nighttime systolic BP levels on top of conventional risk factors and office systolic BP from 0.69 to 3.06%.

Being consistent with previous publications, the prevalence of cerebral microbleeds, lacunes, and enlarged perivascular spaces was 11.9, 8.9, and 7.5%, respectively (13, 30). In comparison to other Hispanic participants residing in or outside of the United States, our prevalence is comparable.s In the Northern Manhattan Study, constituted by ~60–70% of Hispanic participants from the general population, the prevalence of lacunes and cerebral microbleeds was 13.4 and 4.92%, respectively (13, 31). Similarly, in a Mexican-American cohort, the prevalence of lacunes and cerebral microbleeds was 4.3 and 10.3%, respectively (32). In addition, in the Atahualpa Project, which is a population-based study of older adults living in rural areas of Ecuador, the rate of lacunes and cerebral microbleeds was 14.7 and 14%, respectively (33). Of note, the distribution of deep, lobar, and mixed cerebral microbleeds was also similar in our study compared to those cohorts of Hispanics participants from the general population.

Numerous studies documented that nighttime BP carries the most valuable prognostic information in relation to adverse health outcomes (12, 34–36). In line with these studies, we also observed that 24-h and nighttime systolic BP showed the strongest association with CSVD. Of note, the role of 24-h systolic BP might be driven by nighttime BP levels due to the high prevalence of nighttime hypertension (68.1%), which can be explained by the change on the ORs of the 24-h systolic BP from above 1 unit to below 1 unit when accounted for nighttime BP levels. The mechanisms involved in the association between CSVD and nighttime BP might be (i) uncontrolled nocturnal hypertension due to lack of antihypertensive therapy during the nighttime (37), or (ii) inherent pathophysiological mechanisms that increase the nighttime BP (38). Antihypertensive therapy is a major confounder when ambulatory BP is examined as the medication is usually taken during the daytime, making it plausible that the BP-lowering effect of the drugs decreases or wean off at night (37). On the other hand, nocturnal hypertension might result from sympathetic modulation of the nighttime BP, disturbed baroreflex sensitivity, sleep apnea, decreased daytime sodium excretion, nocturnal pressure natriuresis, impaired endothelial function, or all (38). Individuals with nocturnal hypertension usually suffer from comorbidities, such as diabetes, dyslipidemia, or obesity, which substantially increase their cardiovascular risk. Patients at high-risk would benefit from antihypertensive therapy guided by ambulatory BP monitoring.

The role of hypertension in the presence and development of CSVD is irrefutable. High office BP relates to CSVD. Considering our findings, nocturnal hypertension might have a greater impact on the brain microcirculation, predisposing to a higher extent the presence of CSVD. Therefore, it is important to describe the physiopathological changes in the cerebral microcirculation associated with hypertension. Based on its etiology, CSVD can be classified into arteriolosclerosis, cerebral amyloid angiopathy, inherited or genetic, inflammatory and immune-mediated, venous collagenosis, and others (3). The most common types are arteriolosclerosis (or type I) and cerebral amyloid angiopathy (type II), but type I pertains to vascular risk factors commonly associated with aging such as hypertension. The physiopathological vascular changes associated with arteriolosclerosis include fibrinoid necrosis, microaneurysm, hyalinosis, fibrohyalinosis, microatheroma, lipohyalinosis, atherosclerosis, and segmental arterial disorganization. These pathological findings define the functional and structural changes of small arteries associated with hypertension. Therefore, by understanding the possible etiologies in CSVD, the identification of risk factors potentially leads to better diagnostic and preventive strategies. This is clinically valuable as hypertension, which is the main risk factor for arteriolosclerosis CSVD, is a preventable modifiable risk factor and yet, it remains the first cause of cardiovascular and cerebrovascular complications worldwide due to the poor diagnosis, treatment, and control rates (9).

The clinical implications of our study rely on the management of nocturnal hypertension for prevention of CSVD. Given the compelling evidence, hypertension guidelines indicated ambulatory BP monitoring as the state-of-art method to measure BP level (10, 11). Yet, substituting office for ambulatory BP measures in clinical settings remains controversial. CSVD is considered as a subclinical marker of target-organ damage, and based on *The Lancet Commissions* (9), controlling BP is the second avoidable level in the prevention of major cardiovascular events, and loss of quality of life, especially in advanced age. With CSVD being a common result of hypertension-related arteriolosclerosis (3), antihypertensive therapy should be guided by both office and ambulatory BP measures, and should be aimed at nighttime normotension. To date, the superiority of bedtime vs. daytime therapy to control BP remains unclear and only one study has addressed such superiority–the Hygia Chronotherapy Trial (39). The study showed that bedtime medication, as opposed to upon waking, improved ambulatory BP control and diminished the risk of major cardiovascular events by 45%. The findings from the Hygia study should be interpreted with caution as its findings remains under debate (40, 41). Until now, the guidelines do not propose nighttime BP treatment as the main therapy target but aiming at daytime and nighttime BP normotension seems reasonable.

No studies have documented the absolute risk of CSVD according to ambulatory BP categories. Due to the cross-sectional design of our study, we were unable to assess absolute risk. However, the probability (%) derived from adjusted logistic regression modeling may put our findings in perspective. As an example, for the same ambulatory hypertension categories, the probability of having a high accumulation of WMH, lacunes, or cerebral microbleeds was higher than the corresponding office hypertension categories – between 3 and 10%. This information is clinically relevant as current international hypertension guidelines recommend treatment for hypertension based on an absolute risk of cardiovascular diseases (e.g., the Framingham Risk Score) (9). Moreover, the absolute risk also influences the decision-making in treating high-risk individuals as demonstrated by the Systolic blood pressure intervention trial (SPRINT) study, where individuals with a higher cardiovascular risk had more benefits with intense antihypertensive treatment compared to patients at low risk (42). Documenting the absolute risk for CSVD based on BP levels, as well as other risk factors, might guide the decision making process to determine which patients should undergo further cardiovascular assessments.

Daytime BP seemed to be the strongest BP index associated with enlarged perivascular spaces, which agrees with the two previously published studies available in the current literature in patients with stroke (18, 43). We hypothesized that the two mechanisms might explain such association. First, the response of cerebral autoregulatory mechanisms to BP. A surge of the BP beyond the cerebral autoregulatory capacity induces dilatation of cerebral vessels and leads to changes in the permeability of the blood-brain barrier (44, 45). Because daytime BP is higher than nighttime BP, the autoregulatory capacity of the cerebral vessels would be more likely exceeded during the daytime. Subsequent changes in the permeability allow fluid to leaks out and overload in the perivascular space (44). Second, the physiological changes of the amount of fluid within the perivascular spaces throughout the day (46) can be exacerbated by higher daytime BP. These hypothetical mechanisms need to be validated, and longitudinal data are needed to determine whether controlling ambulatory BP level will result in lower perivascular spaces.

### Strengths and Limitations

The strengths of our study included the comprehensive information on cardiovascular risk factors, the availability of assessment for ambulatory BP and MRI scans at the same time point, consideration of multicollinearity among BP measurements by applying the residual method, and the separate analyses of subclinical markers of CSVD. The predictive value of nighttime systolic BP was replicable in three outcomes (WMH, lacunes, and cerebral microbleeds), further strengthening our hypothesis. Nevertheless, the present study should be interpreted within the context of its limitations. First, the cross-sectional design prevented causal inferences. Although we were unable to assess the predictive power of ambulatory BP measurements, numerous studies have documented that ambulatory BP measurements predicted the development and/or progression of cerebral small vessel disease (6, 16, 47). Second, we did not record the BP diary and, therefore, we were unable to adequately address the role of antihypertensive treatment in the association between cerebral small vessel disease and ambulatory BP measurements. Third, the generalizability of these findings is limited to the general population, especially because of the overrepresentation of women in the MAS. Lastly, detection of subclinical markers of CSVD strongly depends on MRI characteristics and the detection criteria used.

## Conclusions

Our study demonstrated that ambulatory BP measurements were associated with subclinical markers of CSVD, and such association was superior to office BP measurements. Nighttime systolic BP was the strongest BP measurement associated with WMH, as well as with lacunes. Nevertheless, we observed that the presence of enlarged perivascular spaces was better related to daytime BP than any other BP measurements. The 24-h ambulatory BP monitoring may identify individuals who are at high risk of major cerebrovascular outcomes. In view of the widely available antihypertensive therapy, physicians should focus on controlling ambulatory BP, especially in high-risk individuals (e.g., elderly patients, subjects with history of cardiovascular disease, or with high burden of cardiovascular risk factors). By preventing progression of brain target-organ damage, the risk of major cerebrovascular disease would be substantially reduced, which improves the quality of life and reduces the disability-adjusted life years lost.

## Data Availability Statement

The raw data supporting the conclusions of this article will be made available by the authors if a pertinent request and scientific motivation is submitted to the Maracaibo Aging Study team. Due to the restrictions based on privacy regulations and informed consent of the participants, data cannot be made freely available in public repository.

## Ethics Statement

The studies involving human participants were reviewed and approved by the Institutional Review Boards of the Cardiovascular Institute at the University of Zulia, Maracaibo and Columbia University, New York. The patients/participants provided their written informed consent to participate in this study.

## Author Contributions

JM, GM, JL, JT, DB, and Z-YZ contributed to the conception and design of the study. JM, GM, CG, RL, CC, GC, ES, and Z-YZ contributed to the acquisition of data. JM, JG, LT, LM, CG, RL, CC, and Z-YZ constructed, organized, and managed the database. JM, LT, DW, DB, and Z-YZ performed the statistical analysis. JM, PV, DB, and Z-YZ wrote the draft of the manuscript. GM, JG, LT, TV, SJ, PV, DB, and Z-YZ wrote sections of the manuscript. All authors contributed to manuscript revision, read, and approved the submitted version.

## Funding

This report was supported by the Gene-Environment Interaction in Cognition in Venezuela Families project founded by the National Institute on Aging National Institutes of Health under Award Numbers R01AG036469, AG056609, and 1 R03AG054186-01 (GM and JT). Internal Funds KU Leuven (STG-18-00379) supported the Research Unit Hypertension and Cardiovascular Epidemiology, Department of Cardiovascular Sciences, Leuven.

## Conflict of Interest

The authors declare that the research was conducted in the absence of any commercial or financial relationships that could be construed as a potential conflict of interest.

## Publisher's Note

All claims expressed in this article are solely those of the authors and do not necessarily represent those of their affiliated organizations, or those of the publisher, the editors and the reviewers. Any product that may be evaluated in this article, or claim that may be made by its manufacturer, is not guaranteed or endorsed by the publisher.
