# Supplementary material for: Subclinical Magnetic Resonance Imaging Markers of Cerebral Small Vessel Disease in Relation to Office and Ambulatory Blood Pressure Measurements
Source: Front Neurol. 2022 Jul 14;13:908260. doi: 10.3389/fneur.2022.908260 (PMC9330602; doi:10.3389/fneur.2022.908260)
Supplement: Supplementary file 1 [file Table_1.doc]

***Frontiers in Neurology - Stroke***

***Supplement Data***

This Appendix formed part of the original submission and has been peer reviewed.
Supplement to: *Subclinical Magnetic Resonance Imaging Markers of Cerebral Small Vessel Diseases in Relation to Office and Ambulatory Blood Pressure Measurements*.

Jesus D. Melgarejo, Gladys E. Maestre, Jose Gutierrez, Lutgarde Thijs, Luis J. Mena, Ciro Gaona, Leendertz Reinier, Joseph H. Lee, Carlos A. Chávez, Gustavo Calmon, Egle Silva, Dong-Mei Wei, Joseph D. Terwilliger, Thomas Vanassche, Stefan Janssens, Peter Verhamme, Daniel Bos*, Zhen-Yu Zhang*.

*Joint last authors who contributed equally.

**Table of Contents**

**Table S1.** Number of Readings in Ambulatory Blood Pressure Recordings by Percentile Ranks p2

**Table S2.** Association of Cerebral Microbleeds by Localization with Office and Ambulatory Systolic Blood Pressure Measurements p3

**Table S3.** Association of Quantitative and Categorical Subclinical Markers of Cerebral Small Vessel Disease with Office and Ambulatory Diastolic Blood Pressure Measurements p4

**Table S4.** Association of Quantitative and Categorical Subclinical Markers of Cerebral Small
Vessel Disease with Office and Ambulatory Systolic Blood Pressure Measurements in Participants without Previous History of Stroke p5

**Table S5.** Association of White Matter Hyperintensities with Office and Ambulatory Systolic Blood Pressure Measurements p6

**Table S6.** Association between Cerebral Microbleeds by Localization and Ambulatory Systolic Blood Pressure Measurements p7

**Table S7.** Association between Quantitative Subclinical Markers of Cerebral Small Vessel Disease and Night-to-day Ratio Additionally Adjustment by 24-h, Daytime, and Nighttime
Blood Pressure Measurements p8

**Table S1**

**. Number of Readings in Ambulatory Blood Pressure Recordings by Percentile Ranks**

| **Time interval** | **5** | **25** | **50** | **75** | **95** |
| --- | --- | --- | --- | --- | --- |
| 24-H | 56 | 66 | 70 | 72 | 81 |
| Daytime | 38 | 46 | 49 | 54 | 67 |
| Nighttime | 14 | 15 | 20 | 21 | 22 |

Values are the number of readings corresponding to the 5th, 25th, 50th, 75th and 95th percentiles across 93 recordings. Daytime ranged from 6 am until 11 pm and nighttime from 11 pm until 6 am. Readings were programmed at 15‑minute intervals during the day and at 30‑minute intervals at night.

**Table S2**

**. Association of Cerebral Microbleeds by Localization with Office and Ambulatory Systolic Blood Pressure Measurements**

| **BP Measurements** | **Lobar Cerebral Microbleeds (n = 25)** | |  | **Deep Cerebral Microbleeds (n = 30)** | |  | **Mixed Cerebral Microbleeds (n = 8)** | |
| --- | --- | --- | --- | --- | --- | --- | --- | --- |
|  | **OR (95% CI)** | ***P* value** |  | **OR (95% CI)** | ***P* value** |  | **OR (95% CI)** | ***P* value** |
| **Unadjusted models** |  |  |  |  |  |  |  |  |
| Office systolic BP | 1.14 (0.97-1.32) | 0.103 |  | 1.26 (1.10-1.45) | 0.001 |  | 1.38 (1.08-1.76) | 0.009 |
| 24-H systolic BP | 1.54 (1.23-1.54) | <0.001 |  | 1.51 (1.20-1.90) | <0.001 |  | 2.09 (1.40-3.15) | <0.001 |
| Daytime systolic BP | 1.55 (1.22-2.20) | <0.001 |  | 1.54 (1.20-1.20) | <0.001 |  | 2.16 (1.40-3.35) | <0.001 |
| Nighttime systolic BP | 1.46 (1.20-1.80) | <0.001 |  | 1.39 (1.14-1.70) | 0.001 |  | 1.85 (1.30-2.63) | <0.001 |
| **Adjusted models** |  |  |  |  |  |  |  |  |
| Office systolic BP | 1.13 (0.92-1.40) | 0.244 |  | 1.17 (0.98-1.39) | 0.078 |  | 1.79 (1.17-2.73) | 0.006 |
| 24-H systolic BP | 1.52 (1.18-2.00) | 0.002 |  | 1.41 (1.09-1.82) | 0.009 |  | 2.25 (1.34-3.78) | 0.002 |
| Daytime systolic BP | 1.52 (1.16-2.00) | 0.003 |  | 1.44 (1.09-1.88) | 0.009 |  | 2.44 (1.38-4.31) | 0.002 |
| Nighttime systolic BP | 1.46 (1.16-1.83) | 0.001 |  | 1.30 (1.04-1.62) | 0.022 |  | 1.83 (1.21-2.77) | 0.004 |

OR denotes odds ratio; BP; blood pressure; Odds ratios are given by each +10 mm Hg increase in the systolic BP. Adjusted models accounted sex, age, education, cephalic circumference, body mass index, high-density serum cholesterol, use of antihypertensive treatment, glomerular filtration rate, and previous history of cardiovascular diseases.

**Table S3**

**. Association of Quantitative and Categorical Subclinical Markers of Cerebral Small Vessel Disease with Diastolic Blood Pressure Measurements**

| **BP Measurements** | **Total Log-WMHs (per 1-SD increase)** | |  | **Presence of Lacunes (n = 38)** | |  | **Presence of Microbleeds (n = 51)** | |  | **Presence of Enlarged  Perivascular Spaces (n = 32)** | |
| --- | --- | --- | --- | --- | --- | --- | --- | --- | --- | --- | --- |
|  | ***β* coefficient (95% CI)** | ***P* value** |  | **OR (95% CI)** | ***P* value** |  | **OR (95% CI)** | ***P* value** |  | **OR (95% CI)** | ***P* value** |
| **Unadjusted models** |  |  |  |  |  |  |  |  |  |  |  |
| Office diastolic BP | 0.05 (0.01-0.09) | 0.018 |  | 0.98 (0.84-1.14) | 0.785 |  | 0.99 (0.87-1.14) | 0.931 |  | 1.15 (0.98-1.36) | 0.095 |
| 24-H diastolic BP | 0.08 (0.03-0.12) | 0.001 |  | 1.13 (0.95-1.35) | 0.181 |  | 1.13 (0.97-1.32) | 0.123 |  | 1.27 (1.05-1.53) | 0.004 |
| Daytime diastolic BP | 0.07 (0.02-0.11) | 0.005 |  | 1.08 (0.90-1.29) | 0.404 |  | 1.11 (0.95-1.30) | 0.190 |  | 1.27 (1.05-1.54) | 0.001 |
| Nighttime diastolic BP | 0.08 (0.04-0.12) | <0.001 |  | 1.18 (1.02-1.37) | 0.027 |  | 1.13 (0.99-1.29) | 0.064 |  | 1.19 (1.01-1.40) | 0.033 |
| **Adjusted models** |  |  |  |  |  |  |  |  |  |  |  |
| Office diastolic BP | 0.05 (0.01-0.08) | 0.021 |  | 0.97 (0.82-1.14) | 0.682 |  | 0.98 (0.85-1.13) | 0.766 |  | 1.20 (0.99-1.46) | 0.060 |
| 24-H diastolic BP | 0.06 (0.01-0.10) | 0.014 |  | 1.13 (0.92-1.38) | 0.255 |  | 1.12 (0.94-1.33) | 0.198 |  | 1.35 (1.08-1.68) | 0.008 |
| Daytime diastolic BP | 0.05 (0.01-0.09) | 0.033 |  | 1.08 (0.88-1.33) | 0.465 |  | 1.10 (0.92-1.31) | 0.286 |  | 1.36 (1.09-1.70) | 0.007 |
| Nighttime diastolic BP | 0.05 (0.02-0.09) | 0.005 |  | 1.17 (0.99-1.39) | 0.072 |  | 1.12 (0.97-1.29) | 0.119 |  | 1.22 (1.02-1.47) | 0.030 |

OR denotes odds ratio; BP; blood pressure; WMH, white matter hyperintensities. *β* coefficients and odds ratios are given by each +10 mm Hg increase in the systolic BP. Adjusted models accounted sex, age, education, cephalic circumference, body mass index, diabetes mellitus, high-density serum cholesterol, use of antihypertensive treatment, glomerular filtration rate, and previous history of cardiovascular diseases.

**Table S4**

**. Association of Quantitative and Categorical Subclinical Markers of Cerebral Small Vessel Disease with Systolic Blood Pressure Measurements in Participants without Previous History of Stroke**

| **BP Measurements** | **Total Log-WMHs (per 1-SD increase)** | |  | **Presence of Lacunes (n = 38)** | |  | **Presence of Microbleeds (n = 51)** | |  | **Presence of Enlarged  Perivascular Spaces (n = 32)** | |
| --- | --- | --- | --- | --- | --- | --- | --- | --- | --- | --- | --- |
|  | ***β* coefficient (95% CI)** | ***P* value** |  | **OR (95% CI)** | ***P* value** |  | **OR (95% CI)** | ***P* value** |  | **OR (95% CI)** | ***P* value** |
| **Adjusted models** |  |  |  |  |  |  |  |  |  |  |  |
| Office systolic BP | 0.08 (0.04, 0.12) | <0.001 |  | 1.14 (0.97-1.32) | 0.094 |  | 1.13 (0.98-1.30) | 0.101 |  | 1.14 (0.96-1.34) | 0.139 |
| 24-H systolic BP | 0.14 (0.09, 0.19) | <0.001 |  | 1.46 (1.16-1.84) | 0.001 |  | 1.40 (1.13-1.74) | 0.002 |  | 1.29 (1.01-1.65) | 0.040 |
| Daytime systolic BP | 0.13 (0.08, 0.19) | <0.001 |  | 1.43 (1.13-1.80) | 0.003 |  | 1.40 (1.14-1.72) | 0.002 |  | 1.32 (1.03-1.69) | 0.032 |
| Nighttime systolic BP | 0.12 (0.07, 0.17) | <0.001 |  | 1.44 (1.17-1.76) | <0.001 |  | 1.35 (1.13-1.62) | 0.001 |  | 1.23 (0.98-1.53) | 0.067 |

OR denotes odds ratio; BP; blood pressure; WMH, white matter hyperintensities. *β* coefficients and odds ratios are given by each +10 mm Hg increase in the systolic BP. Adjusted models accounted sex, age, education, cephalic circumference, body mass index, diabetes mellitus, high-density serum cholesterol, use of antihypertensive treatment, glomerular filtration rate, and previous history of cardiovascular diseases.

**Table S5**

**. Association of White Matter Hyperintensities with Office and Ambulatory Systolic Blood Pressure Measurements**

| **Log-WMH** | ***β* coefficients (95% CI)** | ***P* value** |
| --- | --- | --- |
| Estimates of office systolic BP |  |  |
| adjusted by 24-h systolic BP | 0.04 (-0.01, 0.09) | 0.053 |
| adjusted by daytime systolic BP | 0.05 (0.01, 0.09) | 0.030 |
| adjusted by nighttime systolic BP | 0.05 (0.01, 0.09) | 0.031 |
| Estimates of 24-h systolic BP adjusted by office systolic BP | 0.10 (0.04, 0.20) | 0.002 |
| Estimates of daytime systolic BP adjusted by office systolic BP | 0.09 (0.03, 0.16) | 0.006 |
| Estimates of nighttime systolic BP adjusted by office systolic BP | 0.09 (0.03, 0.14) | 0.001 |

BP; blood pressure; WMH white matter hyperintensities. *β* coefficients are given by +10 mm Hg increase in the systolic BP. Models accounted sex, age, education, cephalic circumference, body mass index, diabetes mellitus, high-density serum cholesterol, use of antihypertensive treatment, glomerular filtration rate, and previous history of cardiovascular diseases. Models including two correlated systolic BP measurements were constructed using the residual method (see Statistical Analysis section).

**Table S6**

**. Association between Cerebral Microbleeds by Localization and Ambulatory Systolic Blood Pressure Measurements**

| **Cerebral Microbleeds** | **Adjusted for  24-hours systolic BP** | |  | **Adjusted for  Daytime systolic BP** | |  | **Adjusted for  Nighttime systolic BP** | |
| --- | --- | --- | --- | --- | --- | --- | --- | --- |
|  | **OR (95% CI)** | ***P* value** |  | **OR (95% CI)** | ***P* value** |  | **OR (95% CI)** | ***P* value** |
| **Lobar Microbleeds (n = 25)** |  |  |  |  |  |  |  |  |
| 24-H systolic BP | NA | NA |  | 1.10 (0.94-1.29) | 0.254 |  | 1.01 (0.93-1.09) | 0.791 |
| Daytime systolic BP | 0.95 (0.80-1.12) | 0.525 |  | NA | NA |  | 1.01 (0.95-1.07) | 0.753 |
| Nighttime systolic BP | 1.03 (0.96-1.10) | 0.411 |  | 1.03 (0.98-1.08) | 0.205 |  | NA | NA |
| **Deep Microbleeds (n = 30)** |  |  |  |  |  |  |  |  |
| 24-H systolic BP | NA | NA |  | 1.01 (0.86-1.20) | 0.933 |  | 1.05 (0.97-1.13) | 0.213 |
| Daytime systolic BP | 1.03 (0.88-1.20) | 0.710 |  | NA | NA |  | 1.04 (0.98-1.09) | 0.212 |
| Nighttime systolic BP | 0.98 (0.93-1.05) | 0.715 |  | 1.00 (0.96-1.05) | 0.940 |  | NA | NA |
| **Mixed Microbleeds (n = 8)** |  |  |  |  |  |  |  |  |
| 24-H systolic BP | NA | NAA |  | 1.02 (0.75-1.40) | 0.916 |  | 1.11 (0.95-1.30) | 0.180 |
| Daytime systolic BP | 1.08 (0.80-1.49) | 0.665 |  | NA | NA |  | 1.08 (0.96-1.22) | 0.175 |
| Nighttime systolic BP | 0.98 (0.86-1.10) | 0.730 |  | 1.01 (0.92-1.10) | 0.891 |  | NA | NA |

OR, odds ratio; BP; blood pressure; NA, no applicable. OR are given by mm Hg unit increase in the systolic BP. Models accounted sex, age, education, cephalic circumference, body mass index, high-density serum cholesterol, use of antihypertensive treatment, glomerular filtration rate, and previous history of cardiovascular diseases.

**Table S7**

**. Association between Quantitative and Categorical Subclinical Markers of Cerebral Small Vessel Disease and Night-to-day Ratio Additionally Adjustment by 24-h, Daytime, and Nighttime Blood Pressure Measurements**

| **Ambulatory BP measurements** | **Total Log-WMHs (per 1-SD increase)** | |  | **Presence of Lacunes (n = 38)** | |  | **Presence of Microbleeds (n = 51)** | |  | **Presence of Enlarged  Perivascular Spaces (n = 32)** | |
| --- | --- | --- | --- | --- | --- | --- | --- | --- | --- | --- | --- |
|  | ***β* coefficient (95% CI)** | ***P* value** |  | **OR (95% CI)** | ***P* value** |  | **OR (95% CI)** | ***P* value** |  | **OR (95% CI)** | ***P* value** |
| **Model 1** |  |  |  |  |  |  |  |  |  |  |  |
| Night-to-day ratio | 0.14 (0.03, 0.26) | 0.015 |  | 1.31 (0.83-2.05) | 0.246 |  | 1.89 (1.12-3.19) | 0.016 |  | 0.94 (0.54-1.63) | 0.835 |
| **Model 2** |  |  |  |  |  |  |  |  |  |  |  |
| Night-to-day ratio | 0.06 (-0.05, 0.18) | 0.288 |  | 1.55 (0.90-2.70) | 0.114 |  | 1.02 (0.63-1.65) | 0.945 |  | 0.82 (0.46-1.44) | 0.487 |
| 24-hour systolic BP | 0.13 (0.07, 0.18) | <0.001 |  | 1.40 (1.10-1.75) | 0.006 |  | 1.38 (1.12-1.71) | 0.003 |  | 1.31 (1.01-1.68) | 0.040 |
| **Model 3** |  |  |  |  |  |  |  |  |  |  |  |
| Night-to-day ratio | -0.05 (-0.20, 0.08) | 0.441 |  | 1.08 (0.57-2.07) | 0.805 |  | 0.74 (0.41-1.34) | 0.327 |  | 0.63 (0.32-1.24) | 0.183 |
| Nighttime systolic BP | 0.13 (0.07-0.20) | <0.001 |  | 1.15 (1.13-1.84) | 0.003 |  | 1.40 (1.12-1.75) | 0.003 |  | 1.33 (1.02-1.74) | 0.033 |

BP denotes blood pressure; WMH white matter hyperintensities; OR, odds ratio. *β* coefficients are given by each 10/5 mmHg increase in the systolic BP level and 0.10 for night-to-day ratio. Models accounted sex, age, education, cephalic circumference, body mass index, diabetes mellitus, high-density serum cholesterol, use of antihypertensive treatment, glomerular filtration rate, and previous history of cardiovascular diseases. Models including 2 correlated systolic BP measurements were constructed, using the residual method (see Statistical Analysis section).
